# Work and Organizational Psychology Looks at the Fourth Industrial Revolution: How to Support Workers and Organizations?

**DOI:** 10.3389/fpsyg.2018.02365

**Published:** 2018-11-28

**Authors:** Chiara Ghislieri, Monica Molino, Claudio G. Cortese

**Affiliations:** Department of Psychology, University of Turin, Turin, Italy

**Keywords:** fourth industrial revolution, Industry 4.0, future of work, working conditions, human-robot interaction, artificial intelligence, future work skills, employment

## Abstract

With rapid advances in technology in several fields of human life, we are entering the Fourth Industrial Revolution (FIR), which is changing the way businesses create value, people do their work and individuals interact and communicate with each other. In this framework, many questions have arisen about how these transformations affect workers, organizations and societies, and Work and Organizational Psychology (WOP) has been called upon to address some of these open issues. In particular, this article focuses on two aspects of the FIR. The first considers the expansion of automation in the workplace and raises questions such as: how is the relationship between workers and technology changing? How is it affecting people’s well-being? How can we expect it to affect employment and equality in the future? The second is related to how job transformation will influence requirements for knowledge and skills; the main question is: which competence profile, considering hard and soft skills, is required and expected in the work of the future? The aim of the present paper is to improve the understanding of some of the major issues that workers and organizations are, or will be, asked to face, by providing information that will be useful to facilitate debate, research and interventions. In the conclusion section, research, and practical implications at organizational, political and institutional levels are discussed.

## Introduction

In the current era, technology is bringing about many changes: the way in which businesses create and capture value, when, where and how people do their work and the ways in which individuals interact and communicate with each other (Schwab, 2016). Together with trends like globalization, an aging population and urbanization, these changes may affect the future of employment and people’s economic, psychological and physical well-being (Bonekamp and Sure, 2015; Bakhshi et al., 2017). In this framework, several compelling questions have arisen about how such changes are altering the dynamics of jobs, workers and organizations, questions that are still open and need to be clarified before they can be answered.

Cloud and mobile computing, big data and machine learning, sensors and intelligent manufacturing, and advanced robotics are among the main types of technology that are leading this transformation. Together these innovations are moving us toward the Fourth Industrial Revolution (FIR) (Schwab, 2016). Industrial revolutions have always been characterized by technological leaps, ever since the very beginning of industrialization: the First dates back to the end of the 18th century with the advent of mechanization based on water and steam; the Second occurred at the beginning of the 20th century with the intensive use of electrical energy to enable mass production; after World War II, the Third Industrial Revolution introduced electronics and information technology to automate production. The FIR is mainly characterized by the advent of Cyber-Physical Systems (CPS) the blending of hardware and software that can interact with humans to complete work), artificial intelligence and machine learning (Baldassari and Roux, 2017). In literature, the term Industry 4.0 (originally Industrie 4.0, introduced in 2011 in Germany to describe a Government sponsored industrial initiative) is also used to refer to the FIR (e.g., Lasi et al., 2014; Vogel-Heuser and Hess, 2016). Schwab (2016) identified three features that distinguish this Revolution from the previous ones: (1) velocity, since this Revolution is evolving at an exponential rather than a linear pace; (2) scope, it is disrupting nearly all industries; and (3) system impact, as it involves changes capable of transforming production, management, and governance.

The existing literature on the FIR focuses mainly on technology and innovations, application fields, disruption, new opportunities and challenges (Lasi et al., 2014; Vogel-Heuser and Hess, 2016; Liao et al., 2017). However, there is little research on the human-psychological aspects associated with this shift and its influence on work systems, organizations and workers’ preparation and well-being (Barley, 2015). Contributions in this field are, in most cases, for or against the technological revolution, aimed at defining optimistic or worrying scenarios for the future of workers and organizations. To date, few studies have contributed to defining and monitoring the current situation in a structured way and those that are available are mainly qualitative studies summarizing the views of opinion leaders (e.g., Bonekamp and Sure, 2015). Thus, the present paper contributes to the literature showing how research and the practical contribution of Work and Organizational Psychology (WOP) is needed to address potential issues associated with the FIR and to support workers and organizations. As a mini review, the paper does not represent a systematic literature review but tries to offer a short overview of the main questions that may arise from a WOP perspective, referring particularly to the most relevant publications in scientific databases, such as Scopus or Web of Science, regarding the possible implications of the FIR for workers. In doing so, this mini review aims to call for more research and interventions in the WOP field. In the paper, first we describe how the innovations of the FIR are changing workers and technology interaction and affecting people’s work, life and health. Moreover, we discuss how these transformations may be helpful or harmful to workers, and whether they can cause fear among employees that may be detrimental to their well-being and to organizational productivity. In the second part, we introduce skill development and learning processes, highlighting how organizations can prepare themselves and their workers to face this Revolution without disruption or harm. Finally, we summarize an agenda for future research and interventions in the WOP field to better support and collaborate with organizations in this transitional phase.

## The Advent of Automation: New Ways of Working and New Kinds of Workers

The idea that industrial robots can take the role of cooperative and supportive tools is part of the FIR paradigm (Weiss et al., 2016). On the one hand, the use of robots in workplaces carries some benefits, such as lower costs, higher quality, improved safety, and environmental protection; the reduction of high-risk jobs is considered an important aspect at a time when fatal accidents at work are still an extremely significant problem. Moreover, people can participate in defining, creating, and maintaining automated systems (Roblek et al., 2016). Nevertheless, from the psychological perspective, some issues have emerged.

### How Is the Interaction Between Workers and Technology Changing?

Since robots and automated machines are now integrated into workplaces, they have become social actors within that very system; social acceptance has therefore become a critical aspect in their design and implementation (Redden et al., 2014). For robots to become team members, individuals must accept them, communicate effectively with them and, most importantly, trust them (Lewis et al., 2018). The allocation of functions to both humans and robots is an area that needs substantial attention. Among the human-robot interaction issues, Cascio and Montealegre (2016) indicated “decreased situation awareness, distrust of automation, misuse, abuse, and disuse, complacency, decrements in vigilance, and negative effects on other facets of human performance” (p. 358).

On the other side, the increasingly widespread use of different kinds of technology to complete work activities and share information may lead to a reduction in human relationships within the workplace, with potential negative consequences in terms of informal learning, organizational commitment, motivation and well-being. In the literature, several studies using the job-demands resources theory have demonstrated the positive role of co-workers’ informal support as a resource capable of reducing costs associated with job demands, supporting the achievement of goals, and stimulating learning and development (Bakker and Demerouti, 2017). Thus, it seems important to identify and promote new opportunities for human interactions within the changing working conditions.

### How Do New Kinds of Technology Affect People’s Well-Being?

Apart from human-robot interaction, the relationship between individuals and technology is changing in several fields, with potential consequences for people’s well-being. In recent years, advances in telecommunications have changed the ways people experience and organize their work and personal life. Several research have highlighted the negative impact of the use of technology (e.g., laptops or smartphones) during leisure time in terms of well-being and work-life integration (e.g., Derks et al., 2014; Ghislieri et al., 2017). Moreover, the risks of addiction to work-related technology for workers and their family well-being have been demonstrated (e.g., Turel et al., 2011; Quinones et al., 2016). The main issue here seems to be how employers and organizations are contributing to this phenomenon by fostering the “always on” approach that requires workers to be always online and available.

Moreover, it has been suggested that the introduction of innovative systems in the workplace could lead to a lack of autonomy and skills that, in turn, can produce stress, demotivation, and counterproductive work behaviors (Cascio and Montealegre, 2016). Furthermore, workers might have an increased feeling of being controlled and a greater sense of oppression, which would foster dissatisfaction, demotivation and ill-being in the long term. In this regard, a study carried out in Germany, involving opinion leaders, emphasized that the demand for greater transparency and visibility of individuals’ performance and an ever-increasing request for data about employees’ work activities and results, collected through digitized processes, raise additional issues relating to personal data protection (Bonekamp and Sure, 2015).

In general, developments in how people do their work and the uncertainty that changes imply may also result in a transformation in the very meaning of work. As we already know, work plays a central role in modern societies, because it provides people not only with economic security but also personal identity and psychological health (Harpaz, 2002; Blustein, 2008). Work satisfies instrumental needs through income and security, and intrinsic needs by maintaining people’s self-esteem and sense of accomplishment through interpersonal relationships and opportunities for development. WOP research should examine whether and how the meaning of work changes under FIR conditions and what effects these changes have on career commitment and psychological health.

### Are Automated Systems Supporting Humans or Taking Over Their Jobs?

The impact of the FIR on occupation is widely debated and represents one of the main open issues. Several jobs are susceptible to computerization and will soon be at risk (Frey and Osborne, 2017) and many predictions appear drastic in terms of structural unemployment and rising inequality in the future. This Revolution may have consequences for both low-skilled and high-skilled workers: university graduates may find themselves threatened by software capable of performing sophisticated decision-making processes, in a persistently challenging environment characterized by the strenuous pursuit of a balance between the educational system and technological evolution (Ford, 2009; Brynjoifsson and McaAfee, 2014; Bonekamp and Sure, 2015).

Nevertheless, there are also some counter-skeptical positions, which argue that the FIR will undeniably eliminate some jobs in the short term, but will represent an opportunity creating benefits for everyone in the long run (Kaplan, 2015; Weldon, 2016). The key lies in how the technological transformation will take place (Weldon, 2016) and how this transitional phase is managed so that it can lead to a future where technology itself will create new jobs, characterized by less hard and repetitive but more intellectual activities, jobs for which the necessary skills need to be developed through investments in retraining (Kaplan, 2015). In other words, the FIR is regarded as a flywheel for the creation of new employment opportunities in the coming decades, with an increasing need for workers with IT skills and specialized technical expertise. Care professions characterized by a high need for empathy seem to be excluded from these considerations (Bonekamp and Sure, 2015).

However, fear of job loss remains an important issue among employees today, since robots and automation might be seen and perceived as competitors. To date, there are few studies in this field, although initial evidence has confirmed that workers might react by opposing automation. For example, in their study on the usability and acceptance of an industrial robotic prototype, Weiss et al. (2016) found that participants expressed fear of being replaced by robots in the future, although the robot was introduced as a cooperative tool.

## Transformation of Jobs and Skills

According to Gorecky (2014), employees play a strategic role in the FIR, since they “will determine the overall production strategy, monitor the implementation of this strategy, and if need be, intervene in the cyber-physical production system” (Gorecky, 2014, in Pfeiffer, 2015, p. 7). However, this requires specific knowledge and qualifications and a new skills paradigm. Among the conditions that are driving the demand for new skill sets are “comprehensive integration and information transparency; increasing automation of production systems; self-management and decision-making by objects; digital communication and interactive management functions; flexibilization of the use of staff” (Ahrens and Spöttl (2015) in Cevik Onar et al., 2018, p. 138). Nevertheless, in the literature the debate on the need for new skills and how these can be upgraded is still in its early stages (Pfeiffer, 2015).

For psychologists, the intersection between learning and new technology is interesting from many points of view: *how do learning processes change in the context of digital immersion? Is Google becoming a substitute for memory? Does technology positively or negatively affect our ability to learn? How do the life-cycles of our knowledge and competence change? Furthermore, which competence profile, considering hard and soft skills, is needed and expected in the future of work?*

This last question is the one on which WOP should focus more, in order to theoretically define and empirically validate models of adequate skills and, above all, provide practical indications to those actors who contribute to the development of knowledge and skills: educational system, training, organizational practices and employment policies. A study which involved more than 500 representatives in industrial companies, found that 86% attributed increasing importance to life-long learning and 76% expected interdisciplinary cooperation to assume growing importance (Fraunhofer and Ingenics, 2014). Further studies have highlighted the growing significance of teamwork, work-life flexibility (Institut für Führungskultur im digitalen Zeitalter [IFIDZ] and FAZ-Institute, 2014), cross-functional management and cooperation, and cross-company partner networking (Porter and Heppelmann, 2014; Bonekamp and Sure, 2015).

Since most forecasts indicate that there will be greater demand for a higher standard of IT competency in the future (Bonekamp and Sure, 2015), this sheds light on the importance of improving knowledge about digital devices, particularly among engineers, and topics such as virtual, augmented and mixed reality, 3D printing and smart factories (Motyl et al., 2017). Nevertheless, a crucial role is also played by soft skills, especially continuous learning, flexibility, the ability to work in multi-functional teams and to deal with complex situations. Today, this is considered a particularly tricky issue since different studies have highlighted that graduates leave university with insufficient soft skills and that in many cases they are not aware of the importance of these skills in the world of work (Alias et al., 2013; Ghislieri, 2017).

## Conclusion

Any attempt to answer the question “How can work and organizational psychologists support workers and organizations at the time of the FIR?,” requires an agenda for research and interventions that can contribute to the identification of policies aimed at maximizing the positive effects for workers and organizations and minimizing the negative consequences. Despite disagreement over the impact of this Revolution on employment, there is a certain consensus about the importance of identifying adequate and different measures to cope with the ongoing transformation, with interventions at micro, meso and macro levels. Specifically, indications are expected from national and European policies on work and education (from the need for new curricula and updating of higher education to the debate about basic income for everybody) and from organizations and trade union policies (Bonekamp and Sure, 2015).

Since the current literature is scarce, research should first of all aim to deepen the understanding of the interconnection between workers, organizations and technology. Longitudinal studies would be particularly helpful in order to examine the effects of technology on people’s performance, well-being and motivation, and understand whether and when these are positive or negative. Nevertheless, at this stage, preliminary evidence is needed in order to direct future research and decisions, and this could also come from cross-sectional studies. Moreover, it might be useful to focus on specific contexts and categories of workers to draw accurate conclusions, using both qualitative and quantitative methods to build up knowledge in this field.

In order to investigate human-robot interaction and acceptance of automation, valuable models have been described in literature. Venkatesh et al. (2003, 2012) introduced the Unified Theory of Acceptance and Use of Technology (UTAUT), able to explain about 70% of the variance in behavioral intention to use a technology. Weiss et al. (2009) proposed the USUS model, a theoretical and methodological framework to evaluate human-robot collaboration considering Usability, Social acceptance, User experience, and Societal impact. Together with ethnography and observations (Cascio and Montealegre, 2016), such models may be useful for leading research on human-technology dynamics; moreover, they may support interventions, providing useful information for designing and implementing collaborative systems.

According to the Future of Jobs Report (Leopold et al., 2016), only 53% of Chief Human Resources Officers surveyed are confident regarding the adequacy of their organization’s workforce strategy to face changes brought about by the FIR. Thus, a stronger collaboration between practitioners in the HR fields and WOP researchers may be useful to define more effective and shared strategies. In general, practical interventions should consider work analysis, team working, selection, training, talent program and performance management. Applied-research in the field of work-related stress needs to be reviewed, including dimensions linked to the use of new kinds of technology during working and leisure time. Communication processes are also of particular importance in order to accompany people in this transitional phase and deal with doubts and fears that may arise; these processes should involve the working population at large, not only workers directly involved in the change. Moreover, specific interventions for leaders and supervisors might be necessary, in order to support them in dealing with the big transformations associated with the FIR; specific research should investigate how their role is changing and what they will need in order to adopt an effective leadership style able to guide the process of change.

As regards training, educators and policymakers play an essential role in preventing competence obsolescence and fostering the continuous updating and development of those skills required by the current and future labor market. In particular, hard and soft skills must be systematically monitored at the end of university courses, to check whether these profiles meet companies’ expectations, and evaluate the effective use of acquired skills in working contexts. Altogether, these measures should foster the creation of synergies between the educational system and employee training (Bonekamp and Sure, 2015). Enhancing digital skills is a target that is also supported by the European Digital Single Market Strategy (Negreiro, 2015), which sustains digital inclusion projects aimed at tackling and reducing the digital divide, according to which there is still a gap between people who have access to specific information technology and people who do not. This gap influences the development of digital skills as well as opportunities to find a job. Finally, career practitioners also play a crucial role, since they can encourage people to strengthen their ability to deal with changes, to develop their employability skills according to the new needs of the labor market, or to reinvest their skills and professional competence in new jobs in those cases where the Revolution has led to them losing their job. More investments in services of this kind are needed in order to improve their efficacy and accessibility.

## Author Contributions

All authors contributed to this work. CG and MM wrote the manuscript receiving substantial input from CG. All authors approved the final version of the manuscript for submission and agreed to be accountable for all aspects of the work.

## Conflict of Interest Statement

The authors declare that the research was conducted in the absence of any commercial or financial relationships that could be construed as a potential conflict of interest.
